# Supplementary material for: A Multimodal Atlas Reveals the Anatomical Distribution of Medium Spiny Neuron Subtypes and a Novel RGS6+ Population in the Primate Striatum
Source: bioRxiv. 2025 Nov 19:2025.11.16.688724. Preprint. [Version 2] doi: 10.1101/2025.11.16.688724 (PMC12667836; doi:10.1101/2025.11.16.688724)
Supplement: Supplement 1 [file NIHPP2025.11.16.688724v2-supplement-1.pdf]

bioRxiv preprint doi: https://doi.org/10.1101/2025.11.16.688724; this version posted November 19, 2025. The copyright holder for this preprint (which was not certified by peer review) is the author/funder, who has granted bioRxiv a license to display the preprint in perpetuity. It is made available under aCC-BY 4.0 International license.

SUPPLEMENTARY MATERIALS

Supplementary Figures 1-5
Supplementary Tables 1-5
Methods
Acknowledgments

bioRxiv preprint doi: https://doi.org/10.1101/2025.11.16.688724; this version posted November 19, 2025. The copyright holder for this preprint (which was not certified by peer review) is the author/funder, who has granted bioRxiv a license to display the preprint in perpetuity. It is made available under aCC-BY 4.0 International license.

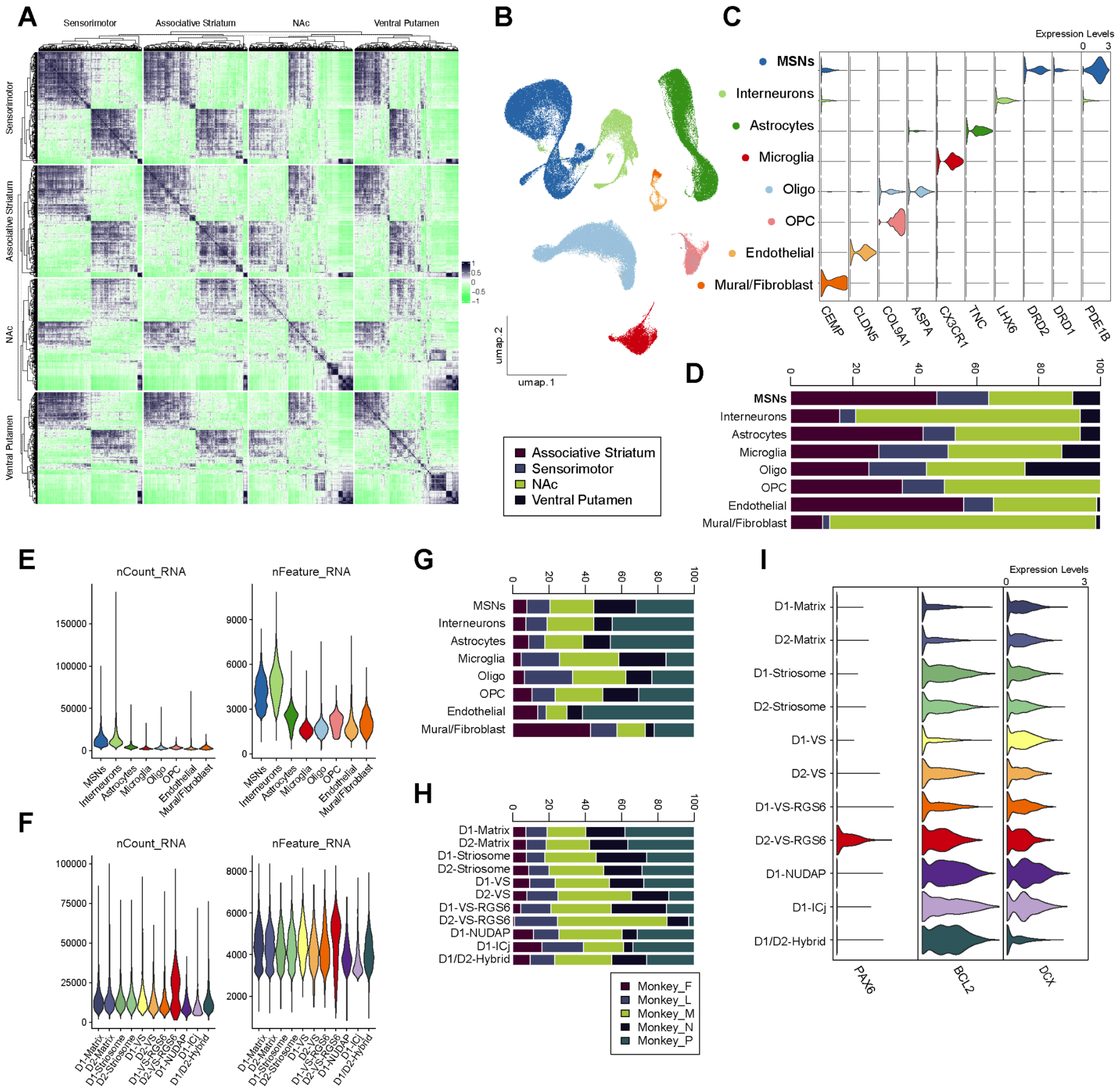

Supplementary Figure 1. Cell Type Distribution Across Subjects-Regions and Quality Control Metrics For snRNA-seq data.

A. Hierarchically clustered heatmap of the gene expression vectors cosine similarity between each pair of cells, rows and columns are split by striatal regions (in A), highlighting transcriptional regional heterogeneity. snRNA-seq dataset was down-sampled to 2000 cells comprising 500 randomly sampled cells per each of the four regions. Cosine similarity was computed between embedding vectors (30 PCs, principal components) per cell.

B. Low dimensionality projection of striatal cell classes (UMAP), after QC filtering, integration of striatal neuronal and non-neuronal nuclei from 4 striatal regions and 5 rhesus macaques, clustering and major cell classes annotations.

C. Violin plots for the major cell classes-specific marker genes neuronal and non-neuronal striatal nuclei. Normalized gene expression per cell type is averaged across regions and animals.

D. Stacked barplot showing the relative regional proportions per cell class for the four functionally defined striatal regions.

bioRxiv preprint doi: https://doi.org/10.1101/2025.11.16.688724; this version posted November 19, 2025. The copyright holder for this preprint (which was not certified by peer review) is the author/funder, who has granted bioRxiv a license to display the preprint in perpetuity. It is made available under aCC-BY 4.0 International license.

E-F. Averaged per-nuclei quality control (QC) metrics across major cell classes (E) and MSNs subtypes (F).
G-H. Stacked barplot showing relative proportions of cells from each of the 5 rhesus macaques per cell class (G) and subclass (H). Cell types are represented across all animals and are not driven by a single subject.
I. Neural progenitor cell markers, *DCX*, *BCL2* and *PAX6* gene expression for the 11 cell subtypes of MSNs. Normalized gene expression per cell type is averaged across regions and animals.

bioRxiv preprint doi: https://doi.org/10.1101/2025.11.16.688724; this version posted November 19, 2025. The copyright holder for this preprint (which was not certified by peer review) is the author/funder, who has granted bioRxiv a license to display the preprint in perpetuity. It is made available under aCC-BY 4.0 International license.

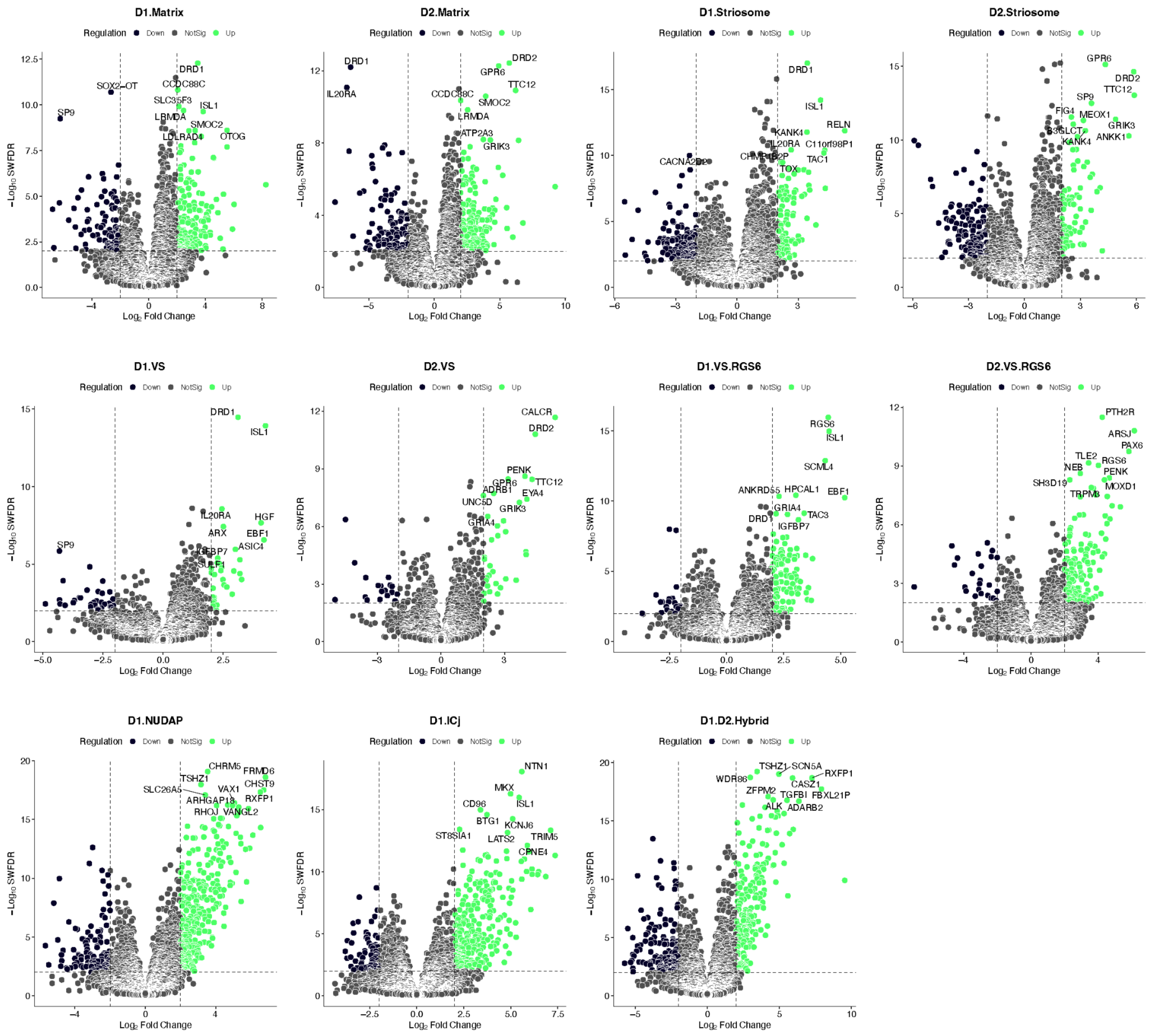

Supplementary Figure 2. Differential Gene Expression Profiles for MSNs Cell Types.

Differential gene expression analysis for each of the eleven identified MSN subtypes in contrast to all other MSN subtypes using Limma-Voom (Supplementary table S1). In each volcano plot, x-axis: Log2(Fold Change), y-axis: negative Log10(Weighted FDR) and the top 10 genes for each cell type are annotated.

bioRxiv preprint doi: https://doi.org/10.1101/2025.11.16.688724; this version posted November 19, 2025. The copyright holder for this preprint (which was not certified by peer review) is the author/funder, who has granted bioRxiv a license to display the preprint in perpetuity. It is made available under aCC-BY 4.0 International license.

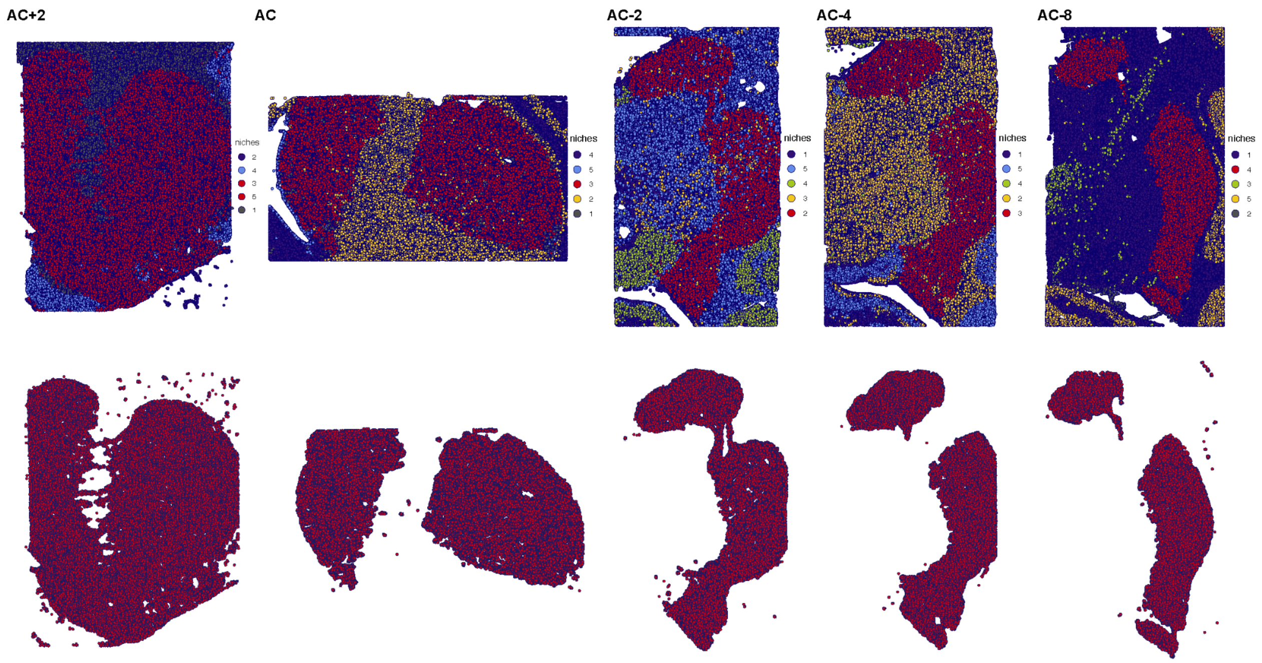

Supplementary Figure 3. Spatial Transcriptomics Striatal Tissue Dissection.
Spatial niche analysis for midbrain tissue sections used for xenium in-situ expression assay across the R-C axis using the anterior Commissure (AC) as the reference level (AC+2, AC, AC-2, AC-4, AC-8). Striatal niche is assigned red color in all sections.

bioRxiv preprint doi: https://doi.org/10.1101/2025.11.16.688724; this version posted November 19, 2025. The copyright holder for this preprint (which was not certified by peer review) is the author/funder, who has granted bioRxiv a license to display the preprint in perpetuity. It is made available under aCC-BY 4.0 International license.

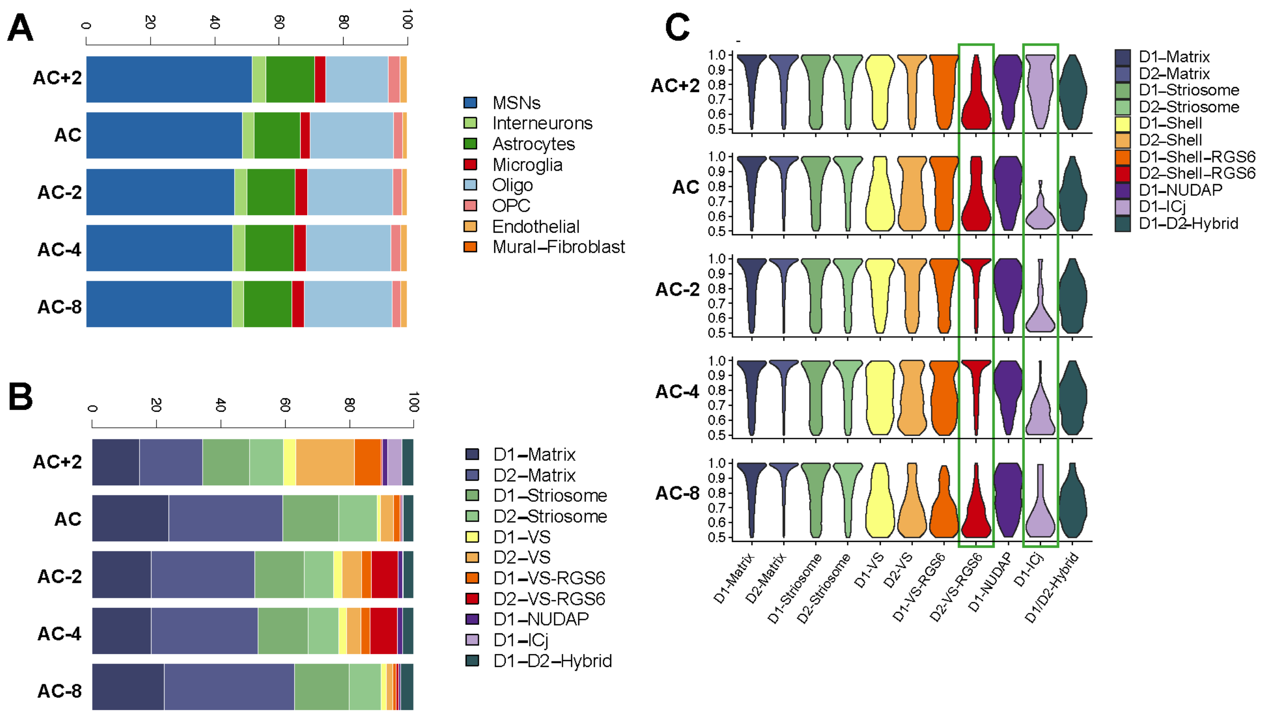

Supplementary Figure 4. Regional Cell Type Prediction and Composition for Spatial Transcriptomics.

A-B. Stacked barplot showing regional-cell type relative proportions of major cell types (A) and MSNs subtypes (B) in five sections across the RC axis.

C. Violin plots of the prediction scores for MSNs subtypes (thresholded at 0.5). Green rectangles highlight the increase in prediction scores depending on the abundance of a cell type, D2-VS-RGS6 in caudal sections and D1-ICj in rostral section.

bioRxiv preprint doi: https://doi.org/10.1101/2025.11.16.688724; this version posted November 19, 2025. The copyright holder for this preprint (which was not certified by peer review) is the author/funder, who has granted bioRxiv a license to display the preprint in perpetuity. It is made available under aCC-BY 4.0 International license.

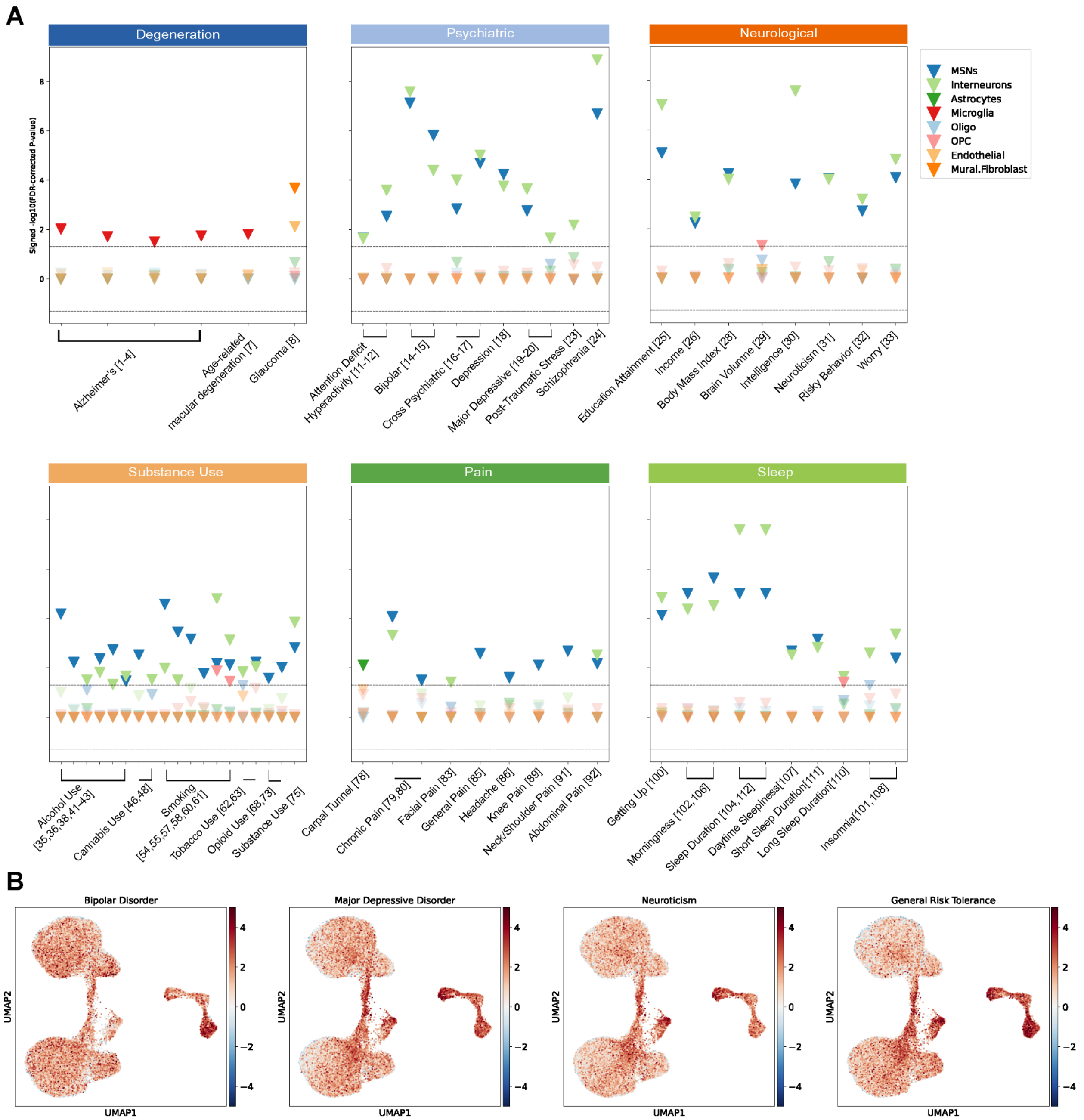

Supplementary Figure 5. Dissecting Polygenic Disease Risk Across Cell Types in the Primate Striatum.

A. Scatterplot for significance of LDSC heritability enrichment across striatal major cell type-Disease pairs. P-values are FDR-adjusted across 11 subtypes and 121 traits, log transformed and multiplied by the sign of the coefficient of association (color scale bar: signed -log10 adjusted p-value). Relevant traits pertaining to neurodegenerative, psychiatric, neurological, sleep and substance use (Table S2). Black dotted line marks FDR=0.05. Bold triangles represent significant associations, while faded triangles represent non-significant associations.

B. Mapping scDRS polygenic enrichment score in individual cells on low dimensionality projection of MSN subtypes (UMAP).
